# Muscle, Ageing and Temperature Influence the Changes in Texture, Cooking Loss and Shrinkage of Cooked Beef

**DOI:** 10.3390/foods9091289

**Published:** 2020-09-14

**Authors:** Rozita Vaskoska, Minh Ha, Zahra Batool Naqvi, Jason David White, Robyn Dorothy Warner

**Affiliations:** 1 School of Agriculture and Food, Faculty of Veterinary and Agricultural Sciences, University of Melbourne, Parkville, VIC 3010, Australia; minh.ha@unimelb.edu.au (M.H.); robyn.warner@unimelb.edu.au (R.D.W.); 2 School of Animal and Veterinary Sciences, Charles Sturt University, Wagga Wagga, NSW 2650, Australia; zanaqvi@csu.edu.au; 3 Office of the Pro Vice Chancellor Research and Innovation, Charles Sturt University, Wagga Wagga, NSW 2650, Australia; jwhite@csu.edu.au

**Keywords:** Warner-Bratzler Shear Force (WBSF), cooking loss, shrinkage, beef, collagen, sarcomere length, tenderness

## Abstract

This study aimed to quantify the effect of muscle, ageing and cooking temperature on the texture, cooking loss and shrinkage of cooked beef. Cuboids from unaged (1 day post mortem) and aged (14 days post mortem) *semitendinosus*, *biceps femoris* and *psoas major* muscles, from both sides of five beef carcasses, were cooked at four different cooking temperatures (50, 60, 70 and 80 °C) for 30 min. and their Warner–Bratzler shear force (WBSF), cooking loss and shrinkage (longitudinal and transverse) were quantified. The WBSF was reduced by ageing in the muscles at the specific cooking temperatures: *psoas major* (cooked at 50, 60 and 80 °C), *semitendinosus* (70 and 80 °C) and *biceps femoris* (80 °C). The cooking loss was 3% greater in aged compared to unaged muscles. The longitudinal shrinkage was greatest in *psoas major* at 80 °C amongst the muscle types and it was reduced by ageing in *psoas major* (70 and 80 °C) and *biceps femoris* (80 °C). The transverse shrinkage was reduced by ageing only in *biceps femoris*, across all temperatures; and the diameter of homogenized fibre fragments from *semitendinosus* and *biceps femoris* was reduced more by cooking at 50 °C in unaged compared to aged condition. WBSF was related to transverse shrinkage, and cooking loss was related to longitudinal shrinkage. The effect of muscle type on the physical changes occurring during cooking of beef is dependent on ageing and cooking temperature.

## 1. Introduction

During cooking, meat shrinks, loses water and changes in tenderness [1]. Meat shrinkage during cooking can be described as a two-dimensional process. Transverse shrinkage, or shrinkage perpendicular to muscle fibre direction, is reported to start between 35 and 45 °C [2,3,4,5,6] and to be completed between 60 and 62 °C [3,4,5,6]. Longitudinal shrinkage, or shrinkage parallel to the direction of the muscle fibres, leading to either sarcomere length or fibre length change, starts between 55 and 64 °C [3,4,5,6] and is completed by 90 °C. Meat shrinkage has been related to the denaturation of myofibrillar and connective tissue proteins in the muscle structure during cooking, and it can be influenced by factors such as ionic strength and pH [2,7,8]. Although the phenomenon of meat shrinkage is widely known and recognised, little research has been conducted on the extent of the shrinkage in muscles with different fibre types, collagen content and sarcomere lengths. Purslow et al. [7] discussed the potential attribution of variations in cooking loss to variations in fibre type composition between muscles, while Astruc et al. [9] showed some variations in the extent of protein denaturation in fibres of different fibre type in the same muscle. Bendall and Restall [3] found that the force required to shorten a meat strip with cooking to 94 °C was greater in *sternomandibularis* muscle compared to *psoas major* muscle, reflecting the difference in their collagen content. On the other hand, comparison between the shrinkage of contracted and stretched samples of bovine *semitendinosus* showed that the sarcomere length affects the extent of shrinkage in both directions [10]. Therefore, differences in fibre type, sarcomere length and collagen content between muscles are expected to affect the shrinkage behaviour of beef.

It is well known that tenderness and juiciness, as well as flavour, define consumer acceptability of beef [11]. Early meat science research tried to establish the connection between the dimensional changes, and the tenderness and cooking loss of meat. Davey and Gilbert [1] defined two phases of toughening in bovine *sternomandibularis*, at temperatures of 40–50 °C and 65–75 °C, and found that the second toughening stage coincided with the longitudinal shrinkage. Similarly, cooking loss has been considered to be a direct result of the shrinkage of the filament lattice [8]. While Davey and Gilbert [1] found that water loss during cooking of bovine *sternomandibularis* closely followed the longitudinal shrinkage of the muscle strips, Purslow et al. [7] associated the consecutive transverse and longitudinal shrinkage, and the subsequent volume shrinkage, with the cooking loss of bovine *semitendinosus*.

Beef is normally stored at refrigeration temperatures to maintain its microbial safety, and the cold storage allows activity of proteolytic enzymes which degrade mostly myofibrillar proteins [12]. The main proteases involved in this process are calpains and cathepsins [12,13,14， 15]. This process of protein degradation results in improved tenderness of meat, and it is known as ageing or cold storage tenderisation. As there is evidence that ageing and cooking temperature can interact in relation to the physical changes of meat shrinkage, texture and cooking loss [7,16], it is important to explore these changes and their relationships in both contexts. While there are multiple studies quantifying meat shrinkage [1,7,17,18], few studies conducted intermuscular comparisons of the physical changes with ageing [19], ageing and cooking [20], and none in relation to shrinkage.

The aim of this study was to understand how ageing and cooking to different temperatures affects the Warner–Bratzler shear force (WBSF), cooking loss and shrinkage of three distinct muscles. The novelty of this study includes the investigation of the longitudinal and transverse shrinkage in muscles of different characteristics and relating the extent of shrinkage to WBSF and cooking loss, which has not previously been reported for *semitendinosus* (silverside, eye of round), *biceps femoris* (outside flat) and *psoas major* (tenderloin). The proposed investigation facilitates a more in-depth understanding of how muscle characteristics affect physical changes during cooking, leading to differences in quality of cooked meat. *Semitendinosus*, *biceps femoris* and *psoas major* muscles were chosen as muscles of interest as they have different tenderness, cooking loss, different fibre type composition, content of connective tissue and sarcomere length. *Semitendinosus* is a tough muscle with a high cooking loss as reported in some studies [21], while *biceps femoris* has been reported as tough or a muscle of intermediate toughness [21,22,23,24] despite the low cooking loss [21]. *Psoas major* is known as the most tender bovine muscle [21] with intermediate cooking loss [21]. *Semitendinosus* is predominately composed of type IIB fibres, *biceps femoris* mostly has type II fibres (IIA and IIB) and less IIB fibres than *semitendinosus*, while *psoas major* is mostly composed of type I fibres [25,26]. In addition, these three muscles differ in the amount of collagen and the type of collagen they contain, the presence of elastin, [27,28,29,30], and in their sarcomere lengths [21].

## 2. Materials and Methods

### 2.1. Muscle Procurement, Allocation to Ageing Treatment, Measurement of pH and Sampling

Ten *semitendinosus*, *biceps femoris* and *psoas major* muscles were excised from both sides of five bovine yearling carcasses, free from hormonal growth promotants, dentition 0–2, with average fat depth at the P8 (over the rump) [31] of 8 mm (SD ± 2.0) and average hot carcass weight of 322 kg (SD ± 45 kg). One muscle from each side of the carcass was randomly allocated to an ageing period of either 1 day post mortem (unaged) or 14 days post mortem (aged), with the muscle from the other side allocated to the alternate ageing treatment. Ageing of 14 days was chosen as it is commonly used for optimising beef eating quality, particularly in Australia, and it relates to the plateau in proteolysis and hence tenderization i.e., little improvement in tenderness after 14 days [32]. Muscle pH was measured in duplicate using a TPS WP 80 pH meter (TPS Pty Ltd., Brisbane, Victoria, Australia) equipped with an IJ-44 electrode (Ionode Pty Ltd., Brisbane, VIC, Australia) and a temperature compensation probe. Thin slices (approximately 50 mm × 10 mm × 3 mm) of the raw muscles were cut parallel to the fibre length for measurement of sarcomere length and a sample of approximately 3 g was taken for determination of collagen content. The samples for sarcomere length were stored at −20 °C while the collagen content samples were stored at −80 °C until measurement.

The muscles allocated to 14 days ageing were stored at 2 °C, after vacuum packaging in polyamide, polyetheylene PA/PE 70 bags (Multivac Sepp Haggenmüller SE & Co. KG, Wolfertschwenden, Germany) with an oxygen permeability <65 cc/m^2^/24 h and water transmission <5 g/m^2^/24 h, using a Multivac C100 vacuum packing machine (Multivac). Sampling for measurement of physical changes was conducted at 1 day post mortem as well as after 14 days of ageing.

### 2.2. Characterization of the Muscles

#### 2.2.1. Sarcomere Length Measurement

Sarcomere length was measured with a laser diffraction unit (custom built by University of New England, Armidale, NSW, Australia). Thin 1-mm slices were shaved from the surface of the frozen sample with a scalpel, placed between two microscope slides and the diffraction distance was measured. The sarcomere length was calculated as in Equation (1):

Sarcomere length = 0.635/SIN(ATAN(X/Y))(1)

where X is the diffraction distance of the sarcomere bands, Y is the calibration distance, measured on the device as the distance of the underside of the diffraction surface and the slide holder, SIN is abbreviation for sine function and ATAN is abbreviation for arctangent function. The sarcomere length of a raw sample from each muscle was calculated as the average of five to eight replicate measurements of the diffraction distance.

#### 2.2.2. Collagen Content Determination

The samples were freeze-dried (Chamber FD3, Dynavac Engineering, Bayswater, VIC, Australia and vacuum pump RV12, Edwards, Burgess Hill, UK) and the collagen content was measured by approximating the amount of hydroxyproline using the method of the Association of Official Agricultural Chemists (AOAC), as described by Starkey et al. [33]. Collagen content is presented only for unaged samples, as collagen is well-known to have little change during ageing post-mortem [34] and also, any changes in collagen post-mortem are not normally reflected in changes in tenderness or shrinkage [35,36].

### 2.3. Physical Changes during Cooking

#### 2.3.1. Method of Cooking, Measurement of Dimensions and Weight

The methods of cooking and measuring dimensions of meat were based on the study of Purslow et al. [7] with slight modifications. Each muscle was cut into twenty (*semitendinosus* and *biceps femoris*) or fifteen cuboids (*psoas major*) with dimensions: length 50 mm × width 30 mm × height 30 mm. The weight was measured with a balance (Ohaus, Parsippany, NJ, USA) before cooking. The 20 (*semitendinosus* and *biceps femoris*) and 15 (*psoas major*) cuboids, four or three, respectively, were randomly assigned to each of the following temperatures: 50 °C, 60 °C, 70 °C and 80 °C and the same number of cuboids was left raw for further measurement and subsampling. A lower number of cuboids per treatment were collected from *psoas major* because of the smaller dimensions of this muscle. A pin was placed on each of the cuboids designated for cooking in the top left corner in order to allow re-alignment of sides to be measured for shrinkage in the three dimensions after cooking. Measurements of dimensions, length, width and height of the cuboid were done with a caliper (Kincrome, Melbourne, Victoria, Australia) of one representative edge before and after cooking. The meat cuboids were placed in individual transparent PE plastic bags and using standard practice [37] the bags were left open. Marbles were used to prevent the bags from floating, as described in the recommended procedure for measuring cooking loss [37]. The bags were then placed in a water bath (Julabo F38; John Morris Scientific, Melbourne, VIC, Australia) pre-heated at one of the treatment temperatures −50, 60, 70 or 80 °C. Samples destined to be treated at the same temperature from both ageing periods and from the three muscle types were placed in the same water bath. The procedure was repeated on the same day for the other three cooking temperatures, and the order of conducting the temperature treatments was randomized across two water baths. Internal temperatures were monitored using temperature T-type thermocouples inserted in the core of the sample (Temperature Controls, Yarraville, VIC, Australia). The samples were cooked for a further 30 min. after the desired internal temperature was reached (Tc). Maximum difference between bath and internal temperature was 0.75 °C. The total cooking time (Tc + 30 min.) was ~ 60 min. Cooking time of ~ 60 min. was used to ensure that changes in cooking loss, shear force and shrinkage are completed, as Locker and Daines [38] have shown that 60–80 min. are needed for these changes to reach a plateau in 40-mm samples of bovine *rectus abdominis*. Fixed holding time (30 min.) after reaching the water bath temperature (Tc) was used, instead of fixed total cooking time, to standardize the cooking method, as a higher water bath temperature would lead to faster heating rate and therefore Tc would vary slightly.

**Figure 1:**
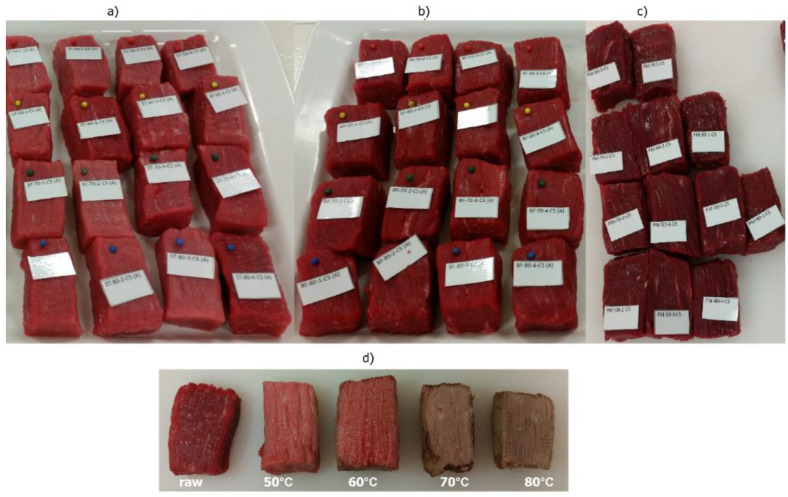
Cuboids from *semitendinosus* (**a**), *biceps femoris* (**b**) and *psoas major* (**c**) cut to 50 mm × 30 mm × 30 mm before cooking. (**d**) Centre of the cuboids from *biceps femoris*, raw and cooked to 50 °C, 60 °C, 70 °C and 80 °C for 30 min (Tc + 30 min.).

Excess water was removed from the cooked meat with paper towel, it was left to cool, and the weight and dimensions of the meat were measured. Figure 1 shows an example of muscle sampling and cooked meat samples from one carcass.

##### Subsampling

The top section of all raw and cooked cuboids (approximately 50 mm × 30 mm × 15 mm) was subsampled and stored overnight at 4 °C for measurement of WBSF. Subsamples of 1 g in total were pooled from the four (*semitendinosus* and *biceps femoris*) and three cuboids (*psoas major*) of the raw and cooked muscles and used for estimation of the diameter of fibre fragments before and after cooking.

#### 2.3.2. WBSF

Two strips (approximately 50 mm × 10 mm × 10 mm) were cut from each sub-sample and WBSF was measured perpendicular to the fibre direction, with a texture analyser (Lloyd; Ametek, Berwyn, PA, USA) using a triangular Warner-Bratzler blade and a speed of 300 mm/min with a load cell of 500 N. The data was obtained from the Nexygen software (Version 3; Bestech, Dingley, VIC, Australia).

#### 2.3.3. Calculation of Cooking Loss, Longitudinal and Transverse Shrinkage

The long dimension of the cuboid was used for the calculation of the longitudinal shrinkage, while for transverse shrinkage, the product of the width and height, Cross-Sectional Area (CSA), was used.

Cooking loss and shrinkage were calculated as the measurements in cooked meat relative to the measurements in raw meat, as in Equation (2).

(2) $$\mathrm{Cooking}\;\mathrm{loss},\;\mathrm{shrinkage}=\frac{\left(\mathrm{weight},\;\mathrm{length},\;\mathrm{CSA}\;\left(\mathrm{raw}\right)\right)-\left(\;\mathrm{weight},\mathrm{length},\;\mathrm{CSA}\;\left(\mathrm{cooked}\right)\right)}{\mathrm{weight},\;\mathrm{length},\;\mathrm{CSA}\;\left(\mathrm{raw}\right)}*100$$

#### 2.3.4. Diameter of Fibre Fragments Obtained by Homogenization of Cooked Meat

Muscle fibre fragments were prepared from the pooled samples of the raw and cooked cuboids, for each treatment within muscle type, ageing period and temperature, according to the method of Purslow et al. [7] with slight modifications. Aliquots of meat (1 g) were prepared by homogenization in 10 mL cold mannitol buffer (380 mM mannitol, 5 mM potassium acetate, pH 5.6) using an Ultra Turrax T25, 10 mm head, (IKA Works, Rawang, Selangor, Malaysia) with a speed of 11,500 rpm. Each sample was homogenised three times for 10 s with 10 s breaks in between homogenisations. A drop of the homogenate was placed on a microscope slide, covered with a cover slip and sealed with nail polish. Observations were made with a compound microscope (Leica DM750, Wetzlar, Germany) and images of muscle fibre fragments were taken with bright field (Leica camera ICC50 W, Wetzlar, Germany) under 200 × magnification (10 × ocular × 20 × objective). At least 15 muscle fibres were measured per muscle, ageing and temperature treatment. If there were less than 15 fibres in the field of vision of one slide, an additional slide was prepared.

### 2.4. Data Analysis

Analysis of pH, collagen and sarcomere length for the muscle characterization was done using Analysis of Variance (ANOVA) in Genstat (Version 18, VSN International, Hemel Hempstead, UK). pH was analysed with muscle and ageing period as fixed factors, while collagen content and sarcomere length were analysed with muscle as fixed factor. Physical measurements (WBSF, cooking loss, longitudinal and transverse shrinkage) were analysed by Restricted Maximum Likelihood Procedure (REML) in Genstat with muscle, aging and temperature as fixed effects and carcass number and side of the carcass as random effects. The WBSF data did not fulfil the assumptions for normal distribution, therefore a logarithmic transformation of the data (base 10) was performed. The diameter was analysed with muscle, ageing and temperature as fixed factors and carcass as random factor. Regression analysis for the relationship between transverse shrinkage and collagen content was conducted in Minitab (Version 19; Minitab, PA, USA). The separation of the data for the relationship between longitudinal shrinkage and sarcomere length was based on the median (2 µm) of the sarcomere length data. Finally, multivariate Principal Component Analysis (PCA) was conducted to identify relationships of the shrinkage to WBSF and cooking loss across the muscles in Minitab.

## 3. Results

### 3.1. Muscle Characterization

Across the three muscle types, the average pH values of the muscles tested were within the pH range for acceptable beef quality (<5.7) [39]. There was a noticeable increase in the pH with ageing in the three muscle types (*p* < 0.01) (Table 1). An increase in pH with ageing has previously been reported for bovine *psoas major* [40] and *longissimus* [41] muscles, and it was associated with a change in charges on the proteins as a result of the proteolytic activity and protein degradation post mortem [41].

As expected, *psoas major* had the longest sarcomere, followed by *semitendinosus* and *biceps femoris* which had comparable sarcomere lengths (Table 2). The sarcomeres of *psoas major* were almost double the length of the sarcomeres of *biceps femoris* (3.45 and 1.88 µm, respectively) (Table 2). The collagen content of the muscles followed an opposite order. *Biceps femoris* had the highest amount of collagen, followed by *semitendinosus,* while *psoas major* had the least amount of collagen (Table 2).

### 3.2. Physical Changes of Bovine Muscles with Ageing and Cooking

#### 3.2.1. WBSF

**Figure 2:**
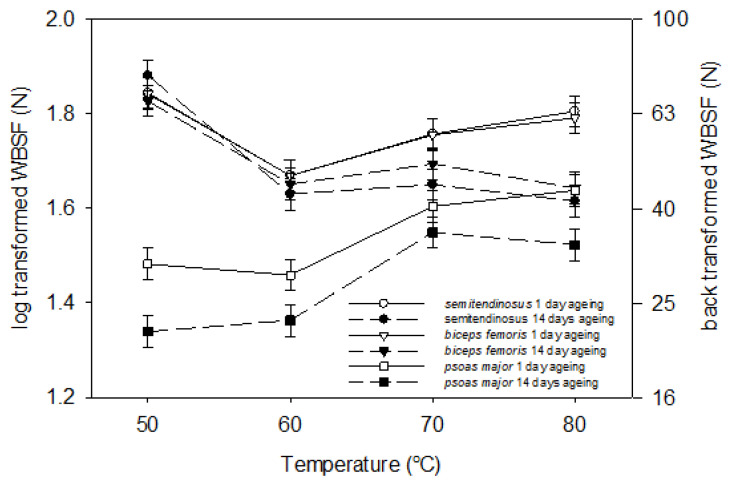
Effect of ageing (1 day post mortem vs. 14 days post mortem) and cooking temperature (50 °C, 60 °C, 70 °C and 80 °C) on Warner–Bratzler shear force (WBSF) of bovine *semitendinosus*, *biceps femoris* and *psoas major*. Cooking time was 30 min. after reaching the defined temperature (Tc + 30 min). Primary y-axis depicts means obtained by analysis of log-transformed data, and secondary y-axis shows back-transformed values of the primary axis. *p* (muscle) < 0.001, *p* (ageing) = 0.006, *p* (temperature) < 0.001, *p* (muscle × ageing) = 0.78, *p* (muscle × temperature) < 0.001, *p* (ageing × temperature) < 0.001, *p* (muscle × ageing × temperature) = 0.013.

Figure 2 illustrates the interaction between muscle, ageing period and temperature (*p* = 0.013) for the WBSF. In most treatments (except when unaged *psoas major* was cooked to 80 °C), *psoas major* had the lowest WBSF amongst the muscles, irrelevant of the treatment (Figure 2). Ageing for 14 days reduced the WBSF of all muscles (*p* < 0.01). *Semitendinosus* and *biceps femoris* had a similar response to ageing and temperature, while *psoas major* showed a distinctly different pattern (Figure 2). Interestingly, when unaged *semitendinosus* and *biceps femoris* were cooked to 70 °C and 80 °C, the WBSF was higher than the WBSF at 60 °C by about 10 N (Figure 2). However, when aged *semitendinosus* and *biceps femoris* were cooked to 60 °C, 70 °C and 80 °C, they remained as tender as when they were cooked at 60 °C (41–49 N) (Figure 2). *Semitendinosus* at 70 °C and 80 °C and *biceps femoris* at 80 °C had greater WBSF cooked as unaged, than when cooked after ageing. The WBSF of *semitendinosus* and *biceps femoris* cooked at 60 °C was 20 N lower than when these muscles were cooked at 50 °C (Figure 2). Unaged *psoas major* had a higher WBSF (by at least 6 N) than aged *psoas major* at all cooking temperatures except at 70 °C where the difference was not significant. The only change in shear force with the increase in temperature in *psoas major* occurred when it was cooked at 70 °C, when the shear force increased by ~12 N compared to 50 °C and 60 °C (Figure 2).

#### 3.2.2. Cooking Loss

**Figure 3:**
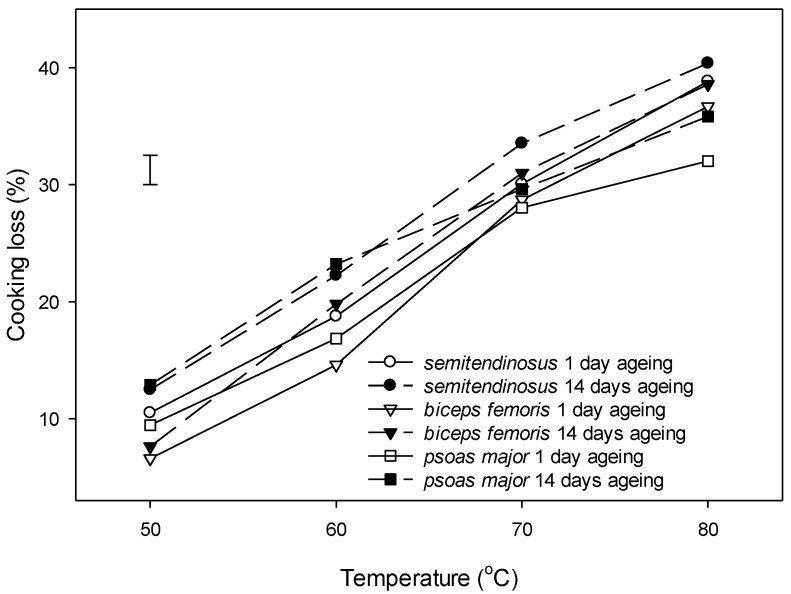
Effect of ageing (1 day post mortem vs. 14 days post mortem) and cooking temperature (50 °C, 60 °C, 70 °C and 80 °C) on cooking loss of bovine *semitendinosus*, *biceps femoris* and *psoas major*. Cooking time was 30 min. after reaching the defined temperature (Tc + 30 min.). Values are least square means and vertical bar on the left is the least significant differences (LSDs) for the three-way interaction muscle × ageing × temperature. Main effects were as follows: *p* (muscle) < 0.001, *p* (ageing) = 0.004, *p* (temperature) < 0.001. Two-way interaction effects were as follows: *p* (muscle × temperature) < 0.001; *p* (ageing × temperature) < 0.001, *p* (muscle × ageing) > 0.05. There was no three-way interaction effect *p* (muscle × ageing × temperature) > 0.05.

There was an interaction between ageing and temperature on cooking loss for all three muscles (*p* < 0.001) (Figure 3). *Semitendinosus* had a significantly greater cooking loss than *biceps femoris* at all cooking temperatures (Figure 3). Aged muscles had a 3% greater cooking loss than unaged muscles across all cooking temperatures and muscles (*p* (muscle) < 0.01). The muscle type and the cooking temperature had a significant interaction effect on the cooking loss (*p* (muscle × temperature) < 0.001). The cooking loss gradually increased with the increase in temperature, and it ranged between 7% and 11% at 50 °C, between 17% and 21% at 60 °C, and between 29 and 32% at 70 °C (Figure 3). At the maximum cooking temperature used in this study 80 °C, the cooking loss was greatest in *semitendinosus* (40%), followed by *biceps femoris* (38%) and *psoas major* (34%).

#### 3.2.3. Shrinkage

**Figure 4:**
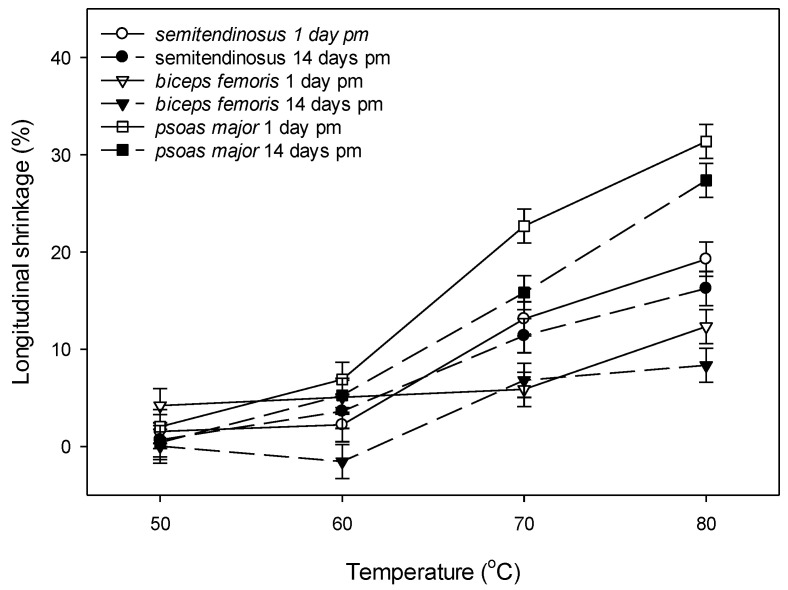
Effect of ageing (1 day post mortem vs. 14 days post mortem) and cooking temperature (50 °C, 60 °C, 70 °C and 80 °C) on longitudinal shrinkage of bovine *semitendinosus*, *biceps femoris* and *psoas major*. Cooking time was 30 min. after reaching the defined temperature (Tc + 30 min.). Values are least square means and vertical bars are least significant differences (LSDs) for the three-way interaction muscle × ageing × temperature. Main and interactive effects were as follows: *p* (muscle) < 0.001, *p* (ageing) = 0.052, *p* (temperature) < 0.001, *p* (muscle × ageing) = 0.063, *p* (muscle × temperature) < 0.001, *p* (ageing × temperature) < 0.633, *p* (muscle × ageing × temperature) = 0.002.

**Figure 5:**
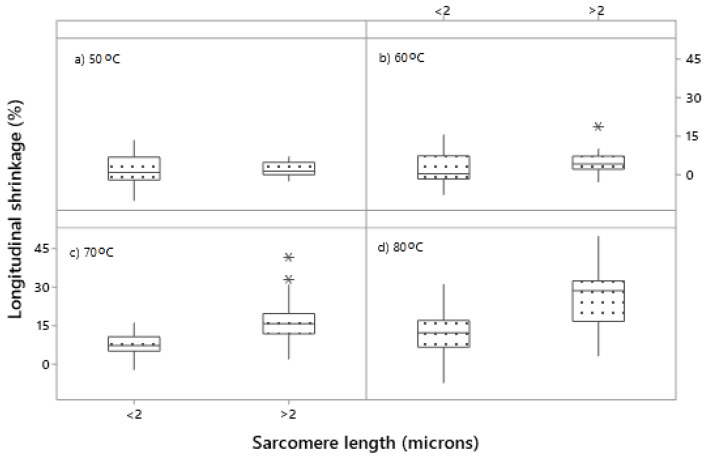
Relationship between longitudinal shrinkage and sarcomere length. Boxplot of the longitudinal shrinkage of muscles with <2 and >2 µm sarcomere length; cooked to (**a**) 50 °C, (**b**) 60 °C, (**c**) 70 °C, (**d**) 80 °C; *n* = 82. A 2-µm threshold was chosen as median of the dataset. Data were combined from three bovine muscles (*semitendinosus*, *biceps femoris* and *psoas major*) from five carcasses. Cooking time was 30 min. after reaching the defined temperature (Tc + 30 min.). Asterisks indicate extreme outliers.

The longitudinal shrinkage was dependent on the interaction between muscle, ageing period and cooking temperature (*p* (muscle × ageing × temperature) < 0.01) (Figure 4). At 50 °C, there was no significant longitudinal shrinkage except for minimal shrinkage in unaged *biceps femoris* (Figure 4). At 60 °C, aged *semitendinosus* and both unaged and aged *psoas major* had their onset of longitudinal shrinkage (the onset calculated as the value exceeding the Least Significant Difference (LSD) of 3.5%), but the shrinkage only ranged between 4% and 7% (Figure 4). At 70 °C and 80 °C, *psoas major* had the greatest longitudinal shrinkage among the muscle types, followed by *semitendinosus* and the least longitudinal shrinkage occurred in *biceps femoris* (Figure 4). There was a trend of a greater longitudinal shrinkage of cuboids from unaged *biceps femoris* (at 50 °C, 60 °C and 80 °C) and *psoas major* (at 70 °C and 80 °C) compared to their aged counterparts (Figure 4). As the magnitude of longitudinal shrinkage corresponded to the sarcomere length across the three muscles, boxplots were used to illustrate the separation in the data, based on the median sarcomere length (2 µm). Muscles with sarcomere length longer than 2 µm had greater longitudinal shrinkage than muscles with sarcomere length shorter than 2 µm when cooked at 70 °C and 80 °C (Figure 5c,d), but not when cooked at lower temperatures.

**Figure 6:**
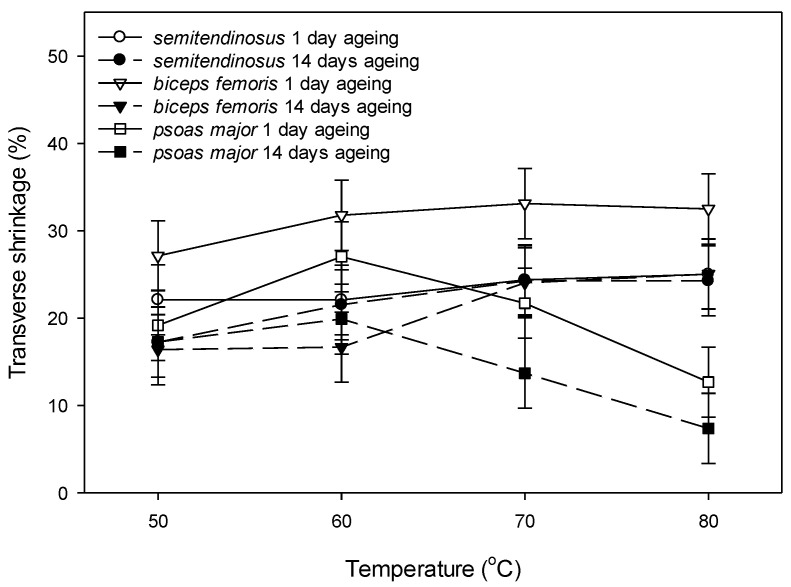
Effect of ageing (1 day post mortem vs. 14 days post mortem) and cooking temperature (50 °C, 60 °C, 70 °C and 80 °C) on transverse shrinkage of bovine *semitendinosus*, *biceps femoris* and *psoas major*. Cooking time was 30 min. after reaching the defined temperature (Tc + 30 min.). Values are least square means and vertical bars are least significant differences (LSDs) for the three-way interaction muscle × ageing × temperature. There was no two-way interactive effect of ageing × temperature nor three-way interaction of muscle × ageing × temperature (*p* > 0.05). Main effects were as follows: *p* (muscle) < 0.001, *p* (ageing) = 0.159, *p* (temperature) = 0.008. Significant interactions were as follows: muscle × temperature *p* < 0.001, and muscle × ageing *p* < 0.001.

**Figure 7:**
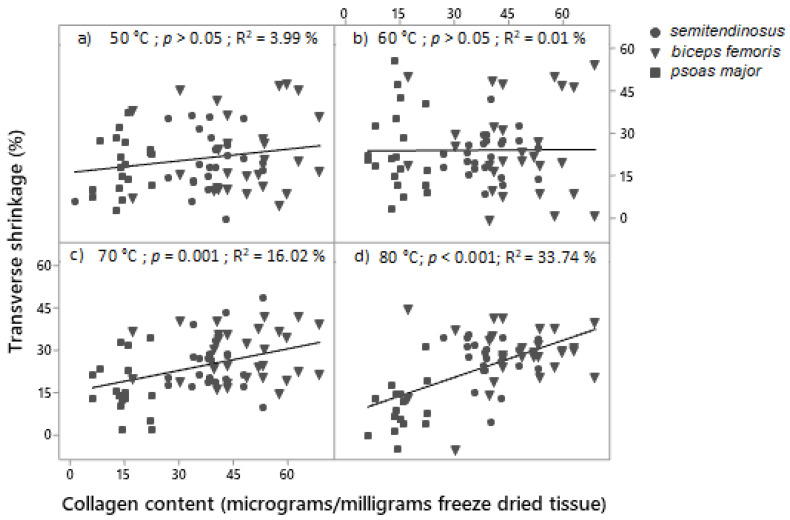
Regression analysis of the relationship between transverse shrinkage and collagen content. Samples cooked at (**a**) 50 °C, (**b**) 60 °C, (**c**) 70 °C, (**d**) 80 °C for 30 min (T_c_ + 30 min.). The *p*-value presents the significance of the regression, R^2^ is percent variance of transverse shrinkage explained by collagen content.

Most of the transverse shrinkage (18–22%) had already occurred at cooking temperature of 50 °C in all muscle types (Figure 6). Ageing period only affected the transverse shrinkage of the cuboids of *biceps femoris* showing a greater transverse shrinkage in unaged than in aged condition (*p* (muscle × ageing) < 0.001) (Figure 6). *Biceps femoris* and *semitendinosus* showed a greater transverse shrinkage at 70 °C compared to 50 °C, and no further increase in transverse shrinkage when cooked to 80 °C (*p* (muscle × temperature) < 0.001) (Figure 6). Interestingly, an increase in transverse shrinkage in *psoas major* from 50 to 60 °C, was followed by a decrease from 60 to 70 °C and 80 °C (Figure 6). Thus, the only significant difference in transverse shrinkage between the muscle types was that at 70 °C and 80 °C, when *psoas major* had the smallest transverse shrinkage relative to the other two muscles. *Biceps femoris* and *semitendinosus* had a greater transverse shrinkage than *psoas major* at when cooked at 80 °C, indicating a potential association between the collagen content of the muscles and the transverse shrinkage, and therefore a regression analysis was conducted as presented in Figure 7a–d. Muscles that had greater collagen content, had greater transverse shrinkage after cooking at 70 °C and 80 °C (Figure 7c,d).

**Figure 8:**
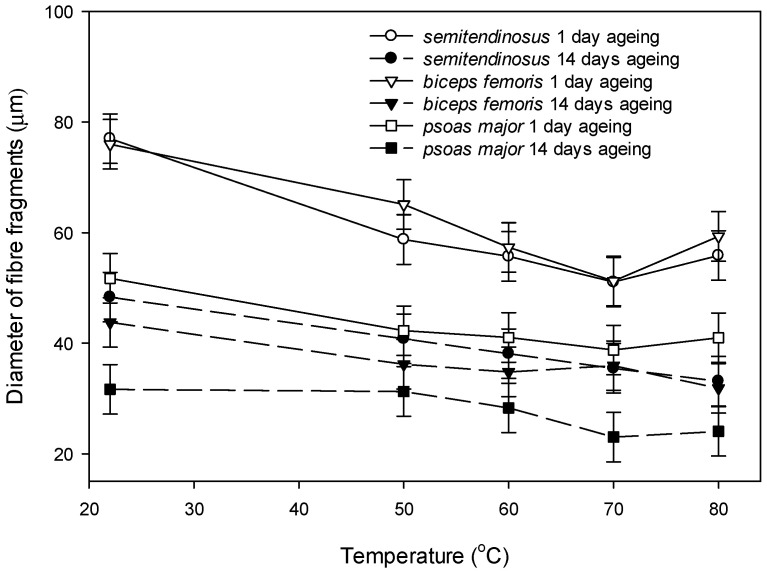
Effect of ageing (1 day post mortem vs. 14 days post mortem) and cooking temperature (raw, 50 °C, 60 °C, 70 °C and 80 °C) on diameter of fibre fragments obtained by homogenization of bovine raw and cooked *semitendinosus*, *biceps femoris* and *psoas major*. Cooking time was 30 min. after reaching the defined temperature (Tc + 30 min.). Values are least square means and vertical bars are least significant differences (LSDs) for the three-way interaction muscle × ageing × temperature. Main and interactive effects were as follows: *p* (muscle) < 0.001, *p* (ageing) < 0.001, *p* (temperature) < 0.001, *p* (muscle × ageing) < 0.001, *p* (muscle × temperature) < 0.001, *p* (ageing × temperature) < 0.001, *p* (muscle × ageing × temperature) = 0.011.

Transverse shrinkage on a fibre level was estimated by observing the diameters of fibre fragments isolated from raw and cooked muscle (Figure 8). There was a three-way interaction of the muscle, ageing period and temperature on the diameter of the fibre fragments isolated from raw and cooked meat (*p* < 0.05). It was evident that muscle fibre fragments from *psoas major* had the smallest diameter amongst the muscles within each temperature treatment of the unaged muscles and in raw aged muscles (Figure 8). The largest change in the diameters of the fibre fragments was the greater reduction in diameter of unaged *semitendinosus* and *biceps femoris* with cooking (10–15 µm reduced diameter), compared to the change in the diameter in the fragments of aged meat with cooking (<10 µm) (Figure 8); which is also evident at the example microscopy images in Supplementary data Figure S1. In relation to temperature, the changes in diameter were more apparent between raw and cooked meat, compared to the changes in diameter of fibre fragments with increasing temperatures (Figure 8).

### 3.3. Multivariate Analysis of the Relationships between Shrinkage and WBSF/Cooking Loss: Ageing and Cooking Temperature

**Figure 9:**
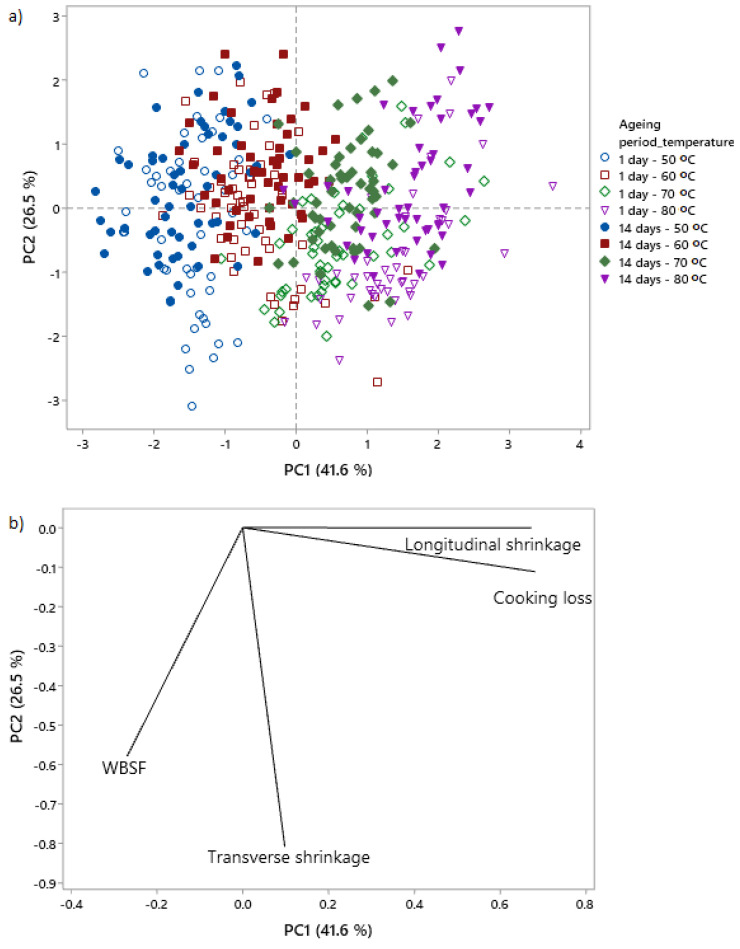
Relationship between the physical changes (WBSF, cooking loss, shrinkage) of bovine *semitendinosus*, *biceps femoris* and *psoas major*. Principal component analysis (PCA) analysis of the physical changes of bovine *semitendinosus*, *biceps femoris* and *psoas major* with ageing and cooking. (**a**) Score plot labelled by ageing period and temperature; (**b**) loading plot corresponding to the PCA analysis in (**a**).

The score plot of the PCA analysis (Figure 9a), in conjunction with the loading plot (Figure 9b) indicated that cooking temperature was the dominating factor affecting the measured variables (WBSF, cooking loss, longitudinal and transverse shrinkage) since the PC1 component explained 41.6% of the variance of the data and it clearly separated the temperature treatments. The score plot (Figure 9a) also illustrates that ageing period is an important factor, since the PC2 component roughly separated the data on ageing period, explaining 26.5% of the variance of the data. The distances between the vectors in the loading plot in Figure 9b demonstrated that longitudinal shrinkage was a greater contributor to cooking loss, and transverse shrinkage contributed more to WBSF. The score plot (Figure 9a) in conjunction with the loading plot (Figure 9b) demonstrate that the increase in temperature is positively correlated to the longitudinal shrinkage and cooking loss, while the ageing of 14 days is correlated to a decrease in WBSF and transverse shrinkage.

## 4. Discussion

### 4.1. Changes in WBSF with Ageing and Cooking

It is well known that meat tenderizes with ageing through disruption of the structure, mainly through proteolysis of myofibrillar proteins such as titin, nebulin and desmin [12]. Cooking related tenderization, on the other hand, is often related to solubilization of collagen. The consistently higher WBSF of unaged *psoas major* at most cooking temperatures, relative to the aged *psoas major*, is interesting and offers evidence that even a very tender muscle, such as *psoas major,* can still undergo tenderization during ageing, although oxidative muscles are normally less prone to proteolysis [42]. The comparable WBSF between ageing periods in *semitendinosus* and *biceps femoris* cooked at 60 °C agrees with the results of Lewis et al. [43] who found the same breaking strength in perimysium of unaged and aged *semitendinosus* when cooked at 60 °C (role of perimysial collagen on WBSF at 60 °C is discussed below). In contrast, for the *semitendinosus* and *biceps femoris* at 70 °C and 80 °C, the increase in WBSF in unaged meat (Figure 2) was likely related to a protein that is prone to proteolysis, therefore changes with ageing, and that also denatures in this temperature region. Two myofibrillar proteins, actin and titin, are known to denature at 78–82 °C [44,45] and 78.4 °C, respectively, [46] and among them, titin can be degraded during ageing [47,48]. Degradation of titin during ageing is a well-established concept and it has been shown that intact titin is degraded by proteolytic enzymes to T1-2 and T2 [48]. The lower WBSF of aged *semitendinosus* and *biceps femoris* compared to unaged muscles at 70 °C and 80 °C is potentially a consequence of the proteolysis of titin during ageing. Degraded titin causes disrupted sarcomeric structure and hence likely results in lower WBSF in aged *semitendinosus* and *biceps femoris* at 70 °C and 80 °C.

It is well-known that cooking temperature influences WBSF. Bouton et al. [49] and Moller et al. [50] associated changes in peak WBSF at 60 °C and 80 °C to changes in connective tissue and myofibrillar proteins, respectively. In general, denaturation and solubilization of connective tissue proteins (collagen) at 60 °C leads to tenderization of meat [51], while denaturation of myofibrillar proteins at 40–54 °C (myosin) and 66–73 °C (actin) leads to toughening of meat [52,53]. Therefore, the reduced WBSF of *semitendinosus* and *biceps femoris* when cooked at 60 °C compared to raw condition and 50 °C (Figure 2) was probably due to denaturation and solubilization of collagen [51], which normally is pronounced with long time cooking [54]. It is logical that collagen solubilization reduces the WBSF of muscles rich in collagen such as *semitendinosus* and *biceps femoris*, but not in muscles low in collagen such as *psoas major* (Figure 2). Christensen et al. [55] also found a similar decrease in tenderness between 50 and 60 °C in bovine *semitendinosus* aged for 2 days and cooked for one hour. It is interesting to note that the change in WBSF with the increase in temperature in *psoas major* in our study (Figure 2) was similar to the modelled change of tensile breaking strength of single muscle fibres of *semitendinosus* in the study of Christensen et al. [55], indicating the importance of the myofibrillar component in the tenderness of *psoas major*.

### 4.2. Changes in Cooking Loss with Ageing and Cooking

Cooking loss of meat is a consequence of protein denaturation caused by heat, with less water held by capillary forces in the structure [56]. The maximum cooking loss of *psoas major* was similar to that previously reported in Hanwoo beef [19]. The lower water-holding capacity of *semitendinosus* and *biceps femoris* than *psoas major* at 80 °C agrees with findings that muscles, predominately of fibre type IIB, retain less water during cooking relative to those with more type I [57]. The cooking loss of *semitendinosus* was consistently than the cooking loss of *biceps femoris* at all cooking temperatures. While the main differences between *semitendinosus* and *biceps femoris* in connective tissue are the greater elastin content [27] and lower amount of collagen I and III in *semitendinosus* [28] compared to *biceps femoris*, it is difficult to attribute the differences in cooking loss to either of these. Elastin is known to be very thermostable up to 80 °C, and starts to denature at temperatures above 80 °C [9]. In addition, the role of different types of collagen in relation to meat texture [30] and cooking loss is not fully understood and requires further investigation. Across the muscles and temperatures, the lowest cooking loss was observed for *psoas major* at 80 °C, suggesting collagen likely plays a role in the shrinkage and eventually in the low cooking loss as *psoas major* had the lowest collagen content. The greater cooking loss of aged compared to unaged bovine muscles has been reported previously for 1 days aged vs. 0 days aged *psoas major* and *semitendinosus* [58]; 14 days vs. 1 day aged *semitendinosus* [7], 14 days vs. 2 days aged *biceps femoris* [32], 14 days vs. 1 day aged *longissimus* [59], and 14 days vs. 2 days aged *longissimus* [60]. In contrast, one study reported similar cooking loss between aged and unaged bovine *semimembranosus* [32]. While it is known that aged meat has a better water holding capacity when measured in raw meat, it appears that the water bound in the raw aged meat is easier to release during cooking in comparison with water in unaged meat. Purslow et al. [7] related this to the degradation of sarcoplasmic proteins during aging that would drag water along with them as they are lost in the expelled juice upon cooking. In addition, the difference in cooking loss between aged and unaged muscle might lie in the chemical nature of the bonds formed between the degraded cytoskeletal proteins and the water during aging, that could be more sensitive to heat. In addition, it has been shown that water in the muscle moves from the intracellular to the extracellular compartments during aging [61] and potentially can be expelled more easily from the structure in aged meat because of its location and because it is not embedded in the filament network. The largest increment of change in cooking loss between 60 and 70 °C differs to the study of Palka and Daun [6] who found the largest increase in cooking loss to be between 50 and 60 °C in bovine *semitendinosus*.

### 4.3. Changes in Shrinkage with Ageing and Cooking

Across the three muscles, the greatest longitudinal shrinkage was found in the *psoas major* and the association between longitudinal shrinkage and sarcomere length (Figure 5) in our study agrees with Dube et al. [62] who found that greater sarcomere length in bovine *psoas major*, resulted in higher sarcomere shortening relative to *longissimus* (which had a shorter sarcomere length) [62]. In other muscles, a similar relationship has been found, where stretched *semitendinosus* has been found to have greater longitudinal shrinkage than cold shortened *semitendinosus* [10]. Lepetit et al. [63] also found a greater longitudinal shrinkage in normal compared to contracted samples of *semimembranosus* and *longissimus dorsi*. The overall longitudinal shrinkage of the muscle cuboids of *psoas major* in our study was greater (~30%) than the 24% sarcomere shortening in the study of Dube et al. [62], as well as compared to the 22.1% shrinkage of stretched *semitendinosus* in the study of Bouton et al. [10]. The smaller longitudinal shrinkage of aged *biceps femoris* and *psoas major* cooked to 80 °C, compared to the unaged samples cooked to the same temperature, indicated a potential role of undegraded proteins such as titin in the process of longitudinal shrinkage. However, we did not find an effect of ageing on the longitudinal shrinkage of cuboids from *semitendinosus* and this agrees with the study of Purslow et al. [7] on the same muscle. In another study we have conducted on a microscopic level, we also found a lower longitudinal shrinkage in fibre fragments from aged compared to unaged *semitendinosus*, *biceps femoris* and *psoas major* when cooked at temperatures >70 °C [64]. The onset of the longitudinal shrinkage at 65–70 °C in our study agrees with previous studies of longitudinal shrinkage in whole meat (*psoas major*, *semitendinosus*) [4,7] and it has been associated with the denaturation of actin [7].

In relation to transverse shrinkage, an interesting phenomenon is the decrease in transverse shrinkage of *psoas major* cuboids at temperatures >60 °C (Figure 6). Two scenarios are possible during cooking of cuboids of *psoas major*: (i) they did not shrink at all in their cross-sectional area (CSA) when cooked at ≥70 °C, or (ii) they shrunk and subsequently expand because of the intense longitudinal shrinkage occurring at these temperatures. These results correspond to the results of Locker and Daines [65] who found 20% swelling in the thickness of strips from *psoas major*. The absence of difference in the transverse shrinkage of unaged and aged cuboids of *semitendinosus* and *psoas major*, coincides with the results of Purslow et al. [7] who did not find any difference with aging in *semitendinosus* cuboids and this is also supported by the work of Latorre et al. [36] who claimed that aging does not affect the thermal shrinkage force of the perimysium in *semitendinosus* (assuming collagen has a role in the process). The greater transverse shrinkage of unaged compared to aged *biceps femoris* can hypothetically be explained by the role of cytoskeletal proteins that might be affected by ageing in the process of transverse shrinkage, assuming that they need to be in undegraded condition to contribute to the transverse shrinkage. Transverse shrinkage was already underway at the lowest temperature used in this study, indicating the important role of myosin denaturation in the transverse shrinkage [7], as myosin is known to denature between 40 and 60 °C [52]. The distinctively greater transverse shrinkage of *biceps femoris* at 50 °C, the consistently greater transverse shrinkage of *biceps femoris* than *semitendinosus*, as well as the lowest transverse shrinkage of *psoas major* at 70 °C and 80 °C, can be associated with the connective tissue content that appears to have a role, in addition to myosin, in the process of transverse shrinkage. This hypothesis, explored visually in Figure 7, warranted further investigation using more extensive characterization of collagen types, solubility, and cross-links, as other authors have found no additional contribution of collagen content to transverse shrinkage over and above myosin [7]. While it is expected that perimysial collagen shrinks longitudinally at 64.5 °C [66], other authors believe that orientation of the collagen fibrils will change in relation to the sarcomere length [63]. Purslow et al. [67] demonstrated that collagen fibrils at longer sarcomere lengths orient at a lower angle compared to shortened fibres where it forms a bigger angle, hence more circumferential orientation. This would imply that the orientation of collagen fibrils in *psoas major* may differ from the orientation in the other two muscles (*semitendinosus* and *biceps femoris*). If collagen orientation is dependent on sarcomere length and has a role in both transverse and longitudinal shrinkage as suggested in some studies [10], it is not surprising that *biceps femoris* has the greatest transverse shrinkage and *psoas major* has the greatest longitudinal shrinkage. However, the contribution of sarcomere length to the longitudinal shrinkage could also be related to a longer I-band which occurs in stretched sarcomeres, allowing more space for filament shrinkage. Other authors have speculated that there is no evidence of the I-band shrinking more than the A-band, therefore questioning the relationship between the I-band length and the longitudinal shrinkage [10]. Alternatively, there are theories for meat shrinkage where collagen is considered not to be a major contributor to meat shrinkage [7] or that it acts as a restraint to the myofibrillar shrinkage [68]. 

### 4.4. Relationships between WBSF, Cooking Loss and Shrinkage as Affected by Ageing and Cooking

The relationship between longitudinal shrinkage and cooking loss has also been previously reported and the concurrent increase in WBSF with the increase in cooking temperature (Figure 9a) agrees with other studies [1]. It is well-known that ageing reduces WBSF as shown in Figure 9a by their association. However, the positive relationship between WBSF and transverse shrinkage does not align with the findings that thinner muscle fibres lead to greater tenderness [69]. On the other hand, the greater transverse shrinkage associated with unaged meat reiterates the findings for *biceps femoris* (Figure 6) and indicates a role of a protein (potentially cytoskeletal) which is affected by ageing for the initiating or conducting of the force driving the observed transverse shrinkage in *biceps femoris*.

## 5. Conclusions

The effect of ageing and cooking at 50 to 80 °C on the WBSF of beef was muscle- and temperature-dependent. *Semitendinosus* and *biceps femoris* showed a similar response, which was distinct from the response of the *psoas major*. Ageing reduced the WBSF of *semitendinosus* and *biceps femoris* at 80 °C, but not at 60 °C while ageing reduced the WBSF of *psoas major* at cooking temperatures of 50 °C, 60 °C and 80 °C. Cooking loss increased with ageing and cooking temperature. Longitudinal shrinkage of the bovine muscles occurred mostly at 70 °C and 80 °C and it was reduced by ageing in *biceps femoris* and *psoas major*. The highest level of transverse shrinkage occurred at the lowest cooking temperature (50 °C), and the transverse shrinkage in *biceps femoris* was reduced by ageing. Muscles with high levels of collagen (*semitendinosus* and *biceps femoris*) had greater transverse shrinkage during cooking, while the *psoas major*, with much longer sarcomere length, showed greater longitudinal shrinkage. Transverse shrinkage was related to the WBSF and longitudinal shrinkage was related to the cooking loss.

## Figures and Tables

**Table 1:** Effect of muscle (*semitendinosus*; *biceps femoris*; *psoas major*) and ageing (1 day post mortem vs. 14 days post mortem) on pH of beef. Values are predicted means.

Muscle	Ageing		SED (Ageing)	*p*-Values		
	1 Day	14 Days		Muscle	Ageing	Muscle × Ageing
*semitendinosus*	5.41	5.49	0.026	>0.05	<0.01	>0.05
*biceps femoris*	5.36	5.50				
*psoas major*	5.48	5.54				

SED—Standard Error of Difference.

**Table 2:** Predicted means for sarcomere length (µm) and total collagen content (µg/mg freeze dried tissue) of bovine *semitendinosus*, *biceps femoris* and *psoas major*.

Characteristic	Muscle	Mean	SED (Muscle)	*p*-Value
Sarcomere length (µm)	*semitendinosus*	2.04	0.059	<0.001
	*biceps femoris*	1.89		
	*psoas major*	3.46		
Total collagen content (µg/mg freeze dried tissue)	*semitendinosus*	39.25	3.969	<0.001
	*biceps femoris*	47.40		
	*psoas major*	14.29		

SED—Standard Error of Difference.

## Supplementary Materials

The following are available online at https://www.mdpi.com/2304-8158/9/9/1289/s1, Figure S1: Example microscopy images of fibre fragments obtained by homogenization of unaged (1 day post mortem) or aged (14 days post mortem) and raw or cooked (to 50 °C, 60 °C, 70 °C and 80 °C) *semitendinosus*, *biceps femoris* and *psoas major* from one carcass. Images obtained at 200× magnification, bar at top left equals 200 µm.
