# Supplementary figures and images for: Muscle, Ageing and Temperature Influence the Changes in Texture, Cooking Loss and Shrinkage of Cooked Beef
Source: Foods. 2020 Sep 14;9(9):1289. doi: 10.3390/foods9091289 (PMC7555138; doi:10.3390/foods9091289)

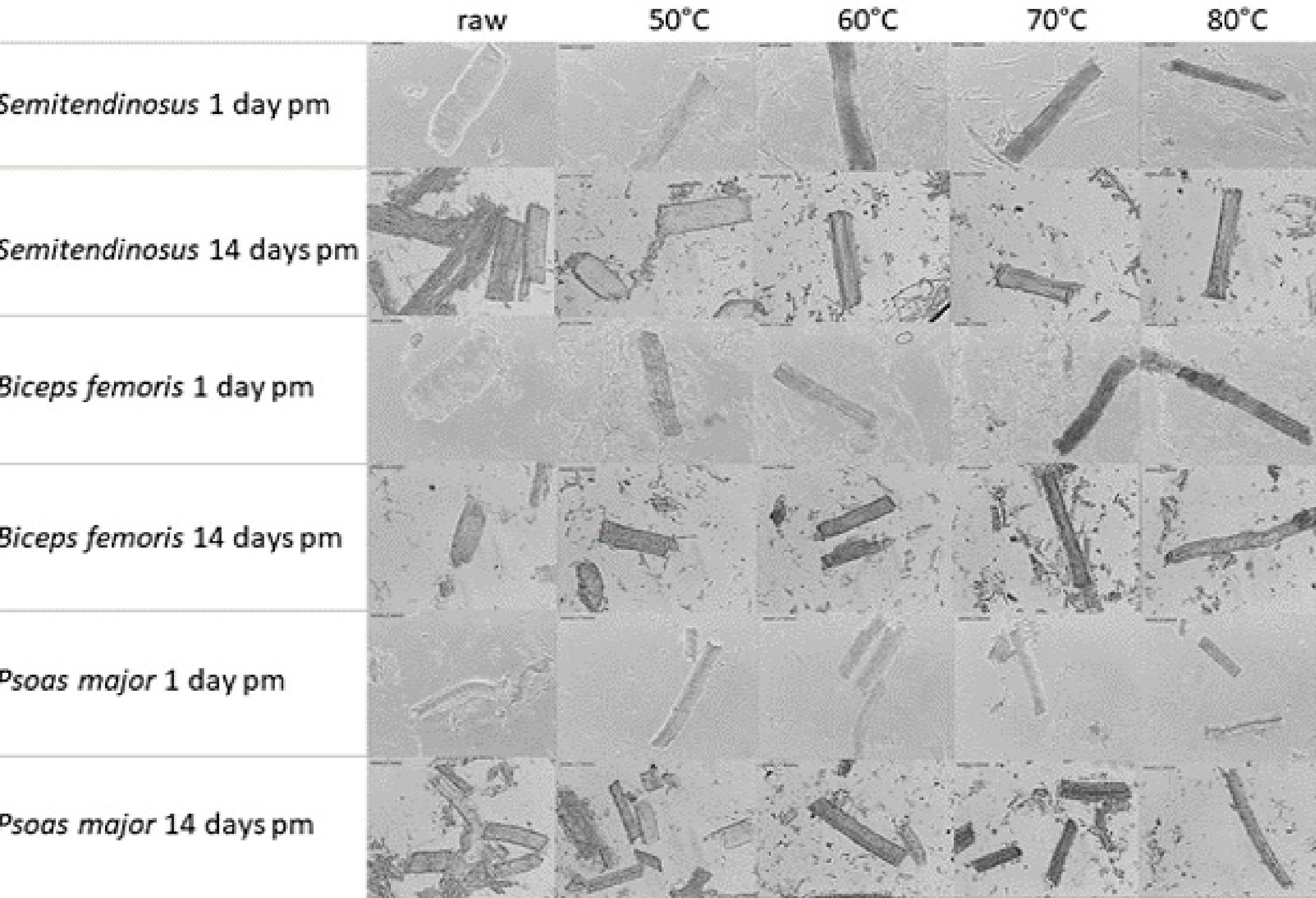

Supplement: Supplementary file 1 [file foods-09-01289-s001.pdf]
